# Utilizing machine learning to analyze trunk movement patterns in women with postpartum low back pain

**DOI:** 10.1038/s41598-024-68798-6

**Published:** 2024-08-12

**Authors:** Doaa A. Abdel Hady, Tarek Abd El-Hafeez

**Affiliations:** 1 Department of Physical Therapy for Women’s Health, Faculty of Physiotherapy, Deraya University, EL-Minia, Egypt; 2 Department of Computer Science, Faculty of Science, Minia University, EL-Minia, Egypt; 3 Computer Science Unit, Deraya University, EL-Minia, Egypt

**Keywords:** Trunk movement, Low back pain, Machine learning, Prediction, Classification, Optuna Regressor, And classifier, Health services, Public health, Quality of life, Therapeutics, Computer science, Information technology, Scientific data

## Abstract

This paper presents an analysis of trunk movement in women with postnatal low back pain using machine learning techniques. The study aims to identify the most important features related to low back pain and to develop accurate models for predicting low back pain. Machine learning approaches showed promise for analyzing biomechanical factors related to postnatal low back pain (LBP). This study applied regression and classification algorithms to the trunk movement proposed dataset from 100 postpartum women, 50 with LBP and 50 without. The Optimized optuna Regressor achieved the best regression performance with a mean squared error (MSE) of 0.000273, mean absolute error (MAE) of 0.0039, and R2 score of 0.9968. In classification, the Basic CNN and Random Forest Classifier both attained near-perfect accuracy of 1.0, the area under the receiver operating characteristic curve (AUC) of 1.0, precision of 1.0, recall of 1.0, and F1-score of 1.0, outperforming other models. Key predictive features included pain (correlation of -0.732 with flexion range of motion), range of motion measures (flexion and extension correlation of 0.662), and average movements (correlation of 0.957 with flexion). Feature selection consistently identified pain, flexion, extension, lateral flexion, and average movement as influential across methods. While limited to this initial dataset and constrained by generalizability, machine learning offered quantitative insight. Models accurately regressed (MSE < 0.01, R2 > 0.95) and classified (accuracy > 0.94) trunk biomechanics distinguishing LBP. Incorporating additional demographic, clinical, and patient-reported factors may enhance individualized risk prediction and treatment personalization. This preliminary application of advanced analytics supported machine learning's potential utility for both LBP risk determination and outcome improvement. This study provides valuable insights into the use of machine learning techniques for analyzing trunk movement in women with postnatal low back pain and can potentially inform the development of more effective treatments.

Trial registration*:* The trial was designed as an observational and cross-section study. The study was approved by the Ethical Committee in Deraya University, Faculty of Pharmacy, (No: 10/2023). According to the ethical standards of the Declaration of Helsinki. This study complies with the principles of human research. Each patient signed a written consent form after being given a thorough description of the trial. The study was conducted at the outpatient clinic from February 2023 till June 30, 2023.

## Introduction

Following childbirth, many women find themselves battling a condition known as postnatal low back pain. This malady can become a chronic issue lasting several months or even beyond, causing discomfort localized to the lower back while also potentially radiating to the legs, buttocks, and hips. Such persistent pain can place a significant burden on a new mother’s life, making the process of caring for her newborn more strenuous^1–3^. The reasons for this condition are multi-rooted. Hormonal changes that transpire during pregnancy and childbirth lead to increased laxity in joints and put an augmented strain on the lower back. Physical transformations that are part and parcel of pregnancy, including weight gain and changes in posture and biomechanics, are also major contributors. Furthermore, psychological factors such as stress, anxiety, and depression may potentiate the onset and prolongation of postnatal low back pain^4,5^.

Several risk factors, such as a history of back pain, a challenging labor and delivery, and inadequate physical activity during pregnancy, are associated with this condition^6^. Women who have undergone cesarean sections might face an elevated risk due to the surgical incision and subsequent changes in biomechanics^7^. Managing postnatal low back pain often necessitates implementing a multidisciplinary approach, including physical therapy, pain management, and psychological support^8^. Physical therapy can aid in strengthening the lower back muscles and ameliorating posture while managing pain through medication and injections can provide relief. Psychological support serves a particularly vital role in equipping new mothers to cope with emotional stressors and psychological challenges, which can compound the manifestations of postnatal low back pain^9^. Statistical estimates suggest that postnatal low back pain is fairly rampant with up to 85% of women likely to experience it during the postpartum period. As one of the most common musculoskeletal disorders, it poses a significant impact on the quality of life of new mothers^10^. However, its prevalence can fluctuate depending on various factors including the woman’s age, number of pregnancies (parity), mode of delivery, and pre-existing musculoskeletal problems. Particularly, women who had multiple pregnancies or are older might face a heightened risk^11^.

In therapy for Low Back Pain (LBP), trunk movement plays a significant role, thereby assessing the trunk’s range of motion (ROM) a critical part of developing rehabilitation programs^12^. Inhibitions in spine mobility can trigger compensatory mechanisms, necessitating enhanced motion from other parts of the body, potentially leading to muscle imbalances, and increasing lumbar issues^13^. Research also hints at differences in the severity of postnatal low back pain, with some women experiencing minor discomfort while others deal with intense pain, disrupting their day-to-day activities. According to one study, about 40% of women with postnatal low back pain reported moderate to severe pain, while the remaining 60% reported experiencing mild to moderate pain. The duration of this condition can also vary significantly. Some women might witness symptoms lasting a few weeks, while it could extend over several months or even longer for others. One survey illustrated that roughly a quarter of women with postnatal low back pain underwent symptoms persisting for over six months^14^.

It’s important to bear in mind that while postnatal low back pain is a pervasive issue, it isn’t universal. Certain factors influence a woman’s susceptibility to developing postnatal low back pain. These factors include pre-existing musculoskeletal conditions, the weight gained during pregnancy, and the mode of delivery, among others. This underlines the criticality of tailoring treatments to the unique needs and circumstances of every woman. Despite its prevalence, understanding the complexity of postnatal low back pain and its multifactorial nature is essential for developing effective strategies for prevention, early detection, and prompt treatment. This understanding could greatly benefit women navigating their postnatal period, playing a tangible role in enhancing their quality of life as they embark on the journey of motherhood. In our study, we are using machine learning models to understand and predict the range of motion (ROM) in the lumbar region. The models are trained with data that includes various factors such as age, height, weight, BMI, VAS for pain, and trunk movements in different directions. By analyzing how these factors relate to ROM, we can identify which ones are the strongest predictors for limitations in ROM. The relationship between limited ROM, particularly in the lumbar region, and lower back pain (LBP) in postpartum women, has been well documented in previous research. Our machine learning models can help corroborate and expand this understanding by accurately predicting lumbar region ROM based on these risk factors. For instance, let’s assume that age and certain trunk movements turn out to be significant predictors for decreased ROM. This information can then potentially be used by healthcare professionals to manage the risk of LBP in postpartum women. If a woman in a certain age group shows certain limitations in trunk movement early on, preventive measures could be applied to help mitigate the risk of developing LBP. In this light, our objective is to apply machine learning to not only understand the risk factors better but also to assist in timely preventive measures that could significantly impact the quality of postpartum care for women.

Our research aims to utilize machine learning models to alleviate lower back pain (LBP) in postpartum women by accurately predicting and classifying the lumbar region Range of Motion (ROM). We understand that this alone cannot attain the comprehensive health improvement mentioned in the statement, such as dietary or routine changes. The intention was to indicate further possible applications of machine learning outside our specific scope. For instance, the application of machine learning extends beyond our research where healthcare practitioners might use technology to analyze individual fitness data, dietary habits, genetic predispositions, and more. Through such extensive data analysis, machine learning can help deliver personalized and proactive healthcare, including the tailoring of exercise regimes, diet plans, and lifestyle changes, although these applications are not tested within our research.

### Problem statement

Despite the high prevalence of postpartum low back pain (LBP) and its significant impact on the quality of life for new mothers, there remains a lack of objective, quantifiable methods for assessing trunk movement patterns associated with LBP. Current diagnostic and treatment approaches are often subjective and do not fully leverage the potential of advanced analytics to personalize interventions. Consequently, there is a critical need for innovative, data-driven strategies that can accurately predict and classify LBP to facilitate more effective management of the condition.

### Research question

Can machine learning algorithms effectively analyze and predict postpartum low back pain in women by classifying trunk movement patterns, and what features are most indicative of abnormal movements associated with LBP?

### Research gap

Previous studies on postpartum LBP have not fully utilized the capabilities of machine learning (ML) techniques to analyze trunk movement data for prediction and classification purposes. There is a research gap in the development and validation of ML models that can accurately differentiate between normal and abnormal trunk movements, identify key predictive features, and contribute to personalized treatment planning for women with postpartum LBP.

### Contributions

1. This study introduces and evaluates machine learning algorithms for the analysis of trunk movement patterns in postpartum women, providing an objective and quantifiable approach to diagnosing and assessing LBP.
2. The research presents a novel application of the Optimized optuna Regressor and Basic CNN in the context of physiotherapy, demonstrating high accuracy in regression and classification tasks for postpartum LBP.
3. By identifying key features associated with LBP, such as pain, flexion, extension, lateral flexion, and average movement, this study contributes to the understanding of biomechanical factors in postpartum women.
4. The development of predictive models that can classify trunk movements offers a potential pathway for personalized treatment plans, helping clinicians tailor interventions based on individual movement patterns.
5. The findings enhance the broader knowledge base regarding the use of advanced analytics in women’s health postpartum, potentially leading to improved outcomes and quality of life for those affected by LBP.

### Related work

In recent years, a rising interest has developed in harnessing advanced machine learning techniques to enrich our comprehension of women’s health in the postnatal phase. Specifically, precise prognosis and categorization of lumbar range of motion have gained significance in evaluating postpartum fitness and overall well-being. Notably, low back pain among postnatal women has been linked to diverse health hazards, including tenderness in the lumbar vertebrae region. Some women experienced postural backache along with discomfort in the sacroiliac joints, particularly aggravated after prolonged sitting. Furthermore, instances were reported where lifting the baby had triggered or exacerbated the pain. The intensity of the pain, which extended down both legs, often necessitated reclining and resting. These issues underscored the potential for postpartum complications^23^. Therefore, the creation of dependable and effective techniques for gauging and foretelling the range of motion (ROM) of the lumbar spine holds paramount significance in advancing women’s health during the postnatal phase. The evaluation of lumbar spine range of motion (ROM) constitutes a crucial element of lumbar spine examination. Irregular spinal motion is linked with flawed spinal mechanics. Lumbar spine movement is evaluated in multiple directions, encompassing flexion, extension, and lateral bending. Typical healthy ranges of motion measured from the anatomical position are as follows: lumbar spine flexion ranges around 52° ± 9°, extension spans 19° ± 9°, while right and left lateral bending reach 31° ± 6°^24^.

Advanced machine learning techniques not only enable prediction but also support the classification of the lumbar region range of motion into various categories or levels of risk. By utilizing supervised classification algorithms like logistic regression, decision trees, and ensemble methods, the lumbar region range of motion can be classified into clinically relevant groups, such as normal, close to normal, and abnormal. These classification models are trained on labeled datasets, where abdominal fat thickness is already categorized based on medical guidelines or expert opinions. After being trained, these models can accurately classify new instances of lumbar region range of motion into the appropriate category, providing valuable information for assessing risk, planning treatment, and monitoring postnatal women’s health^25^.

The utilization of advanced machine learning for the prediction and classification of the lumbar region range of motion brings several benefits to postnatal women’s health. Firstly, these techniques offer enhanced accuracy and precision compared to traditional methods, enabling more reliable assessments of postnatal low back pain. This, in turn, leads to improved risk stratification and personalized interventions tailored to individual needs. Secondly, advanced machine learning algorithms can uncover complex relationships and interactions between various factors contributing to postnatal low back pain. This can provide valuable insights into the underlying mechanisms of postpartum problems and guide the development of targeted interventions for LBP, pelvic pain management, and improved postnatal health.

Table 1 represents a comprehensive summary of critical studies conducted, in recent years, toward understanding and predicting low back pain using various machine learning techniques. Each entry presents an analysis of a unique dataset and algorithm, detailing the year, a summary of the study, the machine learning algorithm employed, and the achieved accuracy. The entries span from 2018 to 2024 and showcase a diverse array of methodologies and outcomes. This array demonstrates the versatility of machine learning algorithms in analyzing different facets of low back pain.

**Table 1:** Summaries of Studies Employing Machine Learning Algorithms for Movement Analysis and Back Pain Assessment.

Authors	Year	Summary	Algorithm	Accuracy
Nait Aicha et al.^26^	2018	Machine learning, applied to accelerometer data gathered from a home environment, provides comparable accuracy to conventional models in identifying fall risks among older individuals. This method’s advantage is its independence from manually extracted features. Ultimately, this technique holds great potential in addressing societal challenges related to promoting active and healthy aging within the comfort of home	CNN, LSTM	(AUC = 0.75)
Abdollahi, et al.^27^	2020	This study tested a combination of a basic, inexpensive motion-capture sensor and the STarT questionnaire to make decisions in healthcare and telemedicine. The results showed that machine learning algorithms, primarily SVM, can differentiate between high-risk and low-risk NSLBP patients with above 75% accuracy. It was also found that tracking trunk movements could partially collect STarT questionnaire data implicitly. These results can help create an objective NSLBP evaluation tool using current technologies, which could have diagnostic and prognostic value. Creating a smartphone app for these tools and sharing quantifiable patient data with doctors, could revolutionize healthcare applications and significantly improve precision medicine	SVM and MLP	Accuracy levels of ~ 75% and 60%
Rothstock, et al.^28^	2020	The study’s key contribution is a clinically useful tool that can help physicians diagnose scoliosis and arrange treatments. The doctor receives assistance in making decisions and designing patient-specific brace treatments according to severity and therapeutic group categorization. The elements of the torso with high asymmetry distances (patches) can be exploited to design unique brace characteristics. Prospects include expanding the patient database and using more advanced neural networks (CNNs) in the context of deep learning	ANNs	90% (SE: 80%, SP: 100%) for curve severity (mild vs. moderate-severe) and 50–72% for the ALS group
Moniri, et al.^29^	2021	Instead of the two lifting strategies used in current cutting-edge research, the best-unsupervised machine learning methodology based on Ward’s clustering correctly discriminated between four separate movement groups in persons with CLBP. The clustering result (four clusters) was confirmed using supervised machine learning using a Bayesian neural network with 97.9% accuracy. This promising method may assist in the more exact evaluation and rehabilitation of people with CLBP	CNN	6.88% mean absolute percentage error and 3.72% standard deviation of absolute percentage error
Phan, et al.^30^	2022	Machine Learning Derived Lifting Techniques and Pain Self-Efficacy in People with Chronic Low Back Pain. *Sensors*	Bayesian neural network	97.9%
Thiry et. al.^31^	2022	The goal of this study was to assess the usefulness of several machine learning algorithms and SampEn in identifying LBP situations. Using raw data from three IMUs and SampEn values collected during a B&R test, the findings demonstrated that the Gaussian NB ML algorithm performed better in discriminating CLBP patients from NLBP subjects than the SampEn discriminant values alone. This study found that supervised ML and a complexity evaluation of trunk variation in motion are beneficial in identifying CLBP situations, while simple kinematic markers are sensitive to the latter	Support Vector Machine, SVM	Achieving 79% accuracy
Zahid Rao^32^	2024	This study showed the creation of an active orthosis for people with impaired trunk control. The use of EMG and IMU data, along with a novel three-level categorization approach, produced encouraging results. Further study into feature selection and model optimization has the potential to improve results, promoting more freedom and well-being for wheelchair users. This study establishes a solid platform for upcoming assistive technologies in trunk rehabilitation	ES and two with KNN	95.44–87.0%
Doaa A. Abdel Hady and Tarek Abd El-Hafee (The Proposed Work)	2024	In this research, machine learning was utilized to analyze and predict low back pain in postnatal women based on trunk movement. A dataset from 100 postpartum women was used, including those with and without low back pain. The optimal regression model was the Optimized optuna Regressor, while the Basic CNN and Random Forest Classifier achieved almost perfect results in pain classification. Key indicators of pain were identified as pain level, range of motion, and average movements. Though the study’s dataset was limited, the machine learning models provided insightful and accurate analysis of the factors contributing to low back pain. The findings underscore the potential of machine learning in improving low back pain risk assessment and tailoring treatment strategies	Optuna, CNN, and Random Forest Classifier	(MSE) of 0.000273, (MAE) of 0.0039, and an R2 score of 0.9968. In classification, the Basic CNN and Random Forest Classifier both attained near-perfect accuracy of 1.0 and F1-score of 1.0,

## Materials

### Trial registration and sample size

This trial was designed as an observational and cross-sectional study. The study was approved by the Ethical Committee in Deraya University, Faculty of Pharmacy, (No: 10/2023). According to the ethical standards of the Declaration of Helsinki, This study complies with the principles for human research. Each patient signed a written consent form after being given a thorough description of the trial. The study was conducted at the outpatient clinic from February 2023 till June 30, 2023.

To prevent type II error, a sample size calculation was performed before the study using the G*Power (Wilcoxon–Mann–Whitney test)^15^, ^16^. The computation was based on statistical markers such as d = 0.5, effect size dz = 0.5, 1−B = 0.95 power analysis, and a 5% significance level on both sides. The computation revealed that a sample size of 40 people was required to ensure that the confidence intervals for the bounds of agreement between any two measures were roughly half a standard deviation of their discrepancies. The dataset size in this study is 60 patients, which is larger than the needed sample size of 40. This means that the study has enough power to identify a significant difference between the two measures with high confidence. As a result, the study has an acceptable sample size, and the results are reliable and genuine.

### Participants and exclusion criteria

The sixty females who were included in the study were diagnosed with postnatal low back pain based on the following criteria: their ages ranged from 25 to 35 years, they were not more than 6 months postnatal, they had three normal deliveries, no postnatal complications for normal cases, and postnatal low back pain for abnormal cases.

Women with a history of disc prolapse and sacroiliac joint problems, symphysis pubic joint as well as lower limb problems, UI and/or lower urinary tract symptoms, neurological diseases, diabetes mellitus, smoking, and cognitive deficit, genital prolapse, leg length discrepancy, diastasis recti, diabetes, intrauterine device, abdominal hernia, and any surgery related to the spine, abdomen, or pelvis.

### Outcome measures

#### Assessment of pain

The agony VAS is a one-dimensional measure of pain intensity that is used to compare the pain severity of patients with similar diseases^17^. When used for acute abdominal pain, it has a high reliability of 0.99 [95% CI 0.989 to 0.992] and a moderate to good reliability for disability in individuals with musculoskeletal pain^18^. With correlations ranging from 0.71 to 0.78 and 0.62 to 0.91, respectively, VAS has been proven to be highly linked with a 5-point verbal descriptive scale (“nil,” “mild,” “moderate,” “severe,” and “very severe”) and a numeric rating scale (with response possibilities ranging from “no pain” to “unbearable pain”)^19^.

#### Body mass index

The body mass index (BMI) for all participants was calculated using the following equation using a universal height-weight scale to ascertain the subject’s height and weight. BMI (Kg/m2): weight (Kg)/Heigh2 (m2), a global classification of BMI values based on a set of cut-off criteria for weight conditions: 18.5 lbs underweight; 18.5–25.0 lbs normal weight; 25.0 lbs overweight^20^.

#### Assessment of lumbar range of movement

**Figure 1:**
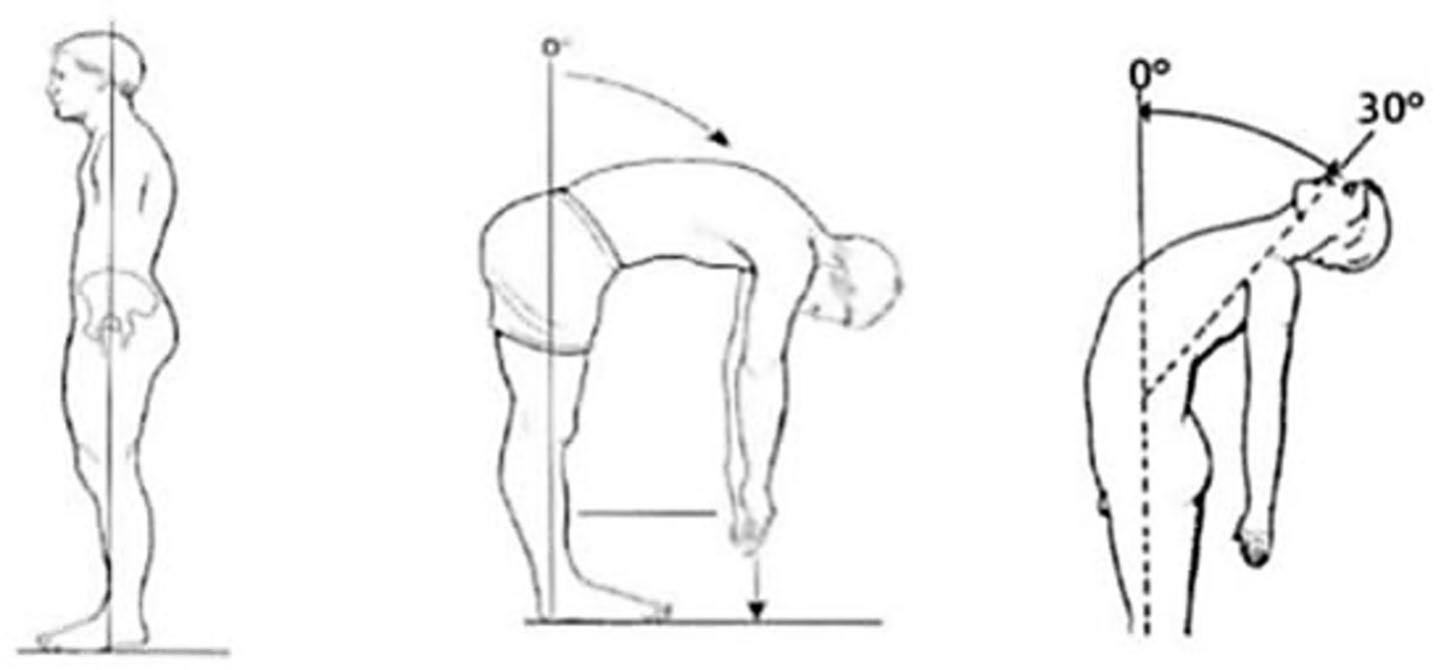
Flexion and extension range of motion (ROM) assessment^21^.

For the examination, a universal goniometer was employed. The active range of motion (ROM) of the trunk was gauged using the methodologies proposed by Norkin and White^21^ and Chertman et al.^22^ for the movements of flexion and extension as depicted in Fig. 1. The participants initiated the test by standing upright, their knees fully straightened, and their arms held forth in front of their bodies. Following oral directives from the examiner, they executed slow, progressive movements for flexion and extension until the maximum amplitude was achieved, at which point the goniometer recorded the measurement. To gauge lumbar flexion, the arms were bent at 90 degrees, but for assessing lumbar extension, participants had to hold their arms behind their bodies. The iliac crest served as the immovable reference point for the measurements, while the adjustable point that was used aligned with the axillary line local to the anterior iliac crest. They ensured that the goniometer’s fixed arm remained centrally located in the lateral region of the trunk during the measurements.

**Figure 2:**
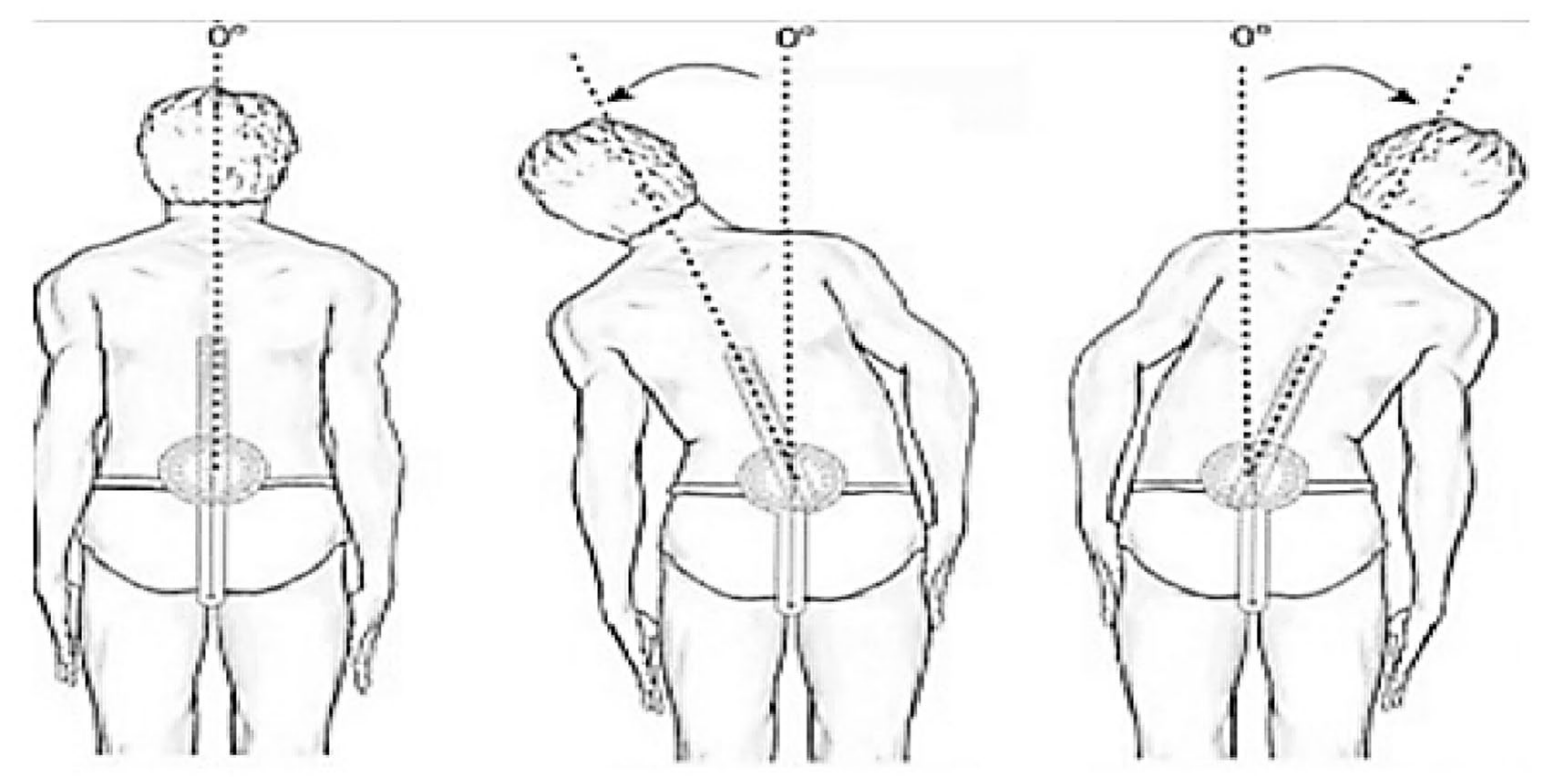
Lateral flexion ROM assessment^21^.

The participants conducted the trunk lateral flexion test in an upright posture with their knees completely extended. Under the guidance of the examiner, they partook in slow, gradual movements of lateral flexion to their fullest amplitude, which was the moment when the goniometric measurement was recorded^21^. The axis-fixed reference point for the lateral flexion examination was the first sacral vertebra. In this setup, the goniometer’s stationary arm was kept perpendicular to the ground, while the movable arm was directed towards the C7 spinous process, as illustrated in Fig. 2.

The same examiner conducted three assessments of the active range of motion for trunk flexion, extension, lateral flexion (both directions), and rotation (both directions). The study utilized the averages of the data collected from each movement. Upon completion of the measurement, the participant was instructed to revert to their initial position, after which the universal goniometer was detached and prepared for the next assessment.

### Ethical Statement

"All procedures performed in studies involving human participants were by the ethical standards of the institutional and/or national research committee and with the 1964 Helsinki Declaration and its later amendments or comparable ethical standards."

## Methodology

After conducting our analysis, we have identified four distinct categories for the lumbar region range of motion classification. These categories include: normal range of motion for flexion is greater than 65 degrees, and abnormal range is equal to or less than 65 degrees. Extension range of motion is classified according to BMI, with less than 25.5 being considered normal and greater than 15 being abnormal. For individuals with a BMI of 29.8 or less, an extension range of motion less than or equal to 18 is abnormal. Additionally, the right lateral flexion of the trunk is considered abnormal when it is less than or equal to 24 degrees, and the left lateral flexion of the trunk is also considered abnormal when it is less than or equal to 24 degrees. These categories provide a standardized framework for evaluating the severity of postnatal low back pain. However, it is important to note that different experts may use alternative ranges or cutoff points. Moreover, the classification system may exhibit variations based on the population under study and other influential factors such as ethnicity, age, and gender.

### Data collection

Our dataset comprises essential variables, namely “Age, Height, Weight, BMI, and VAS. The motion of the lumbar spine is assessed in all planes including flexion, extension, and later flexion bending. Which play a pivotal role in accurately evaluating postnatal low back pain. These variables enable healthcare professionals to effectively assess the health status of women following childbirth, identify individuals with a heightened risk of experiencing adverse health outcomes, and subsequently implement targeted interventions to enhance their overall well-being. Through precise measurements of these variables, healthcare providers gain valuable insights that contribute to informed decision-making and the development of tailored strategies aimed at improving postnatal health outcomes for women. The dataset is available at^33^.

Our selection of machine learning algorithms was based on their suitability for addressing the specific objectives of our study on postnatal low back pain (LBP). The breakdown of our rationale:

1. Optimized Optuna Regressor: We opted for this approach to leverage the Optuna framework’s capability in efficiently exploring a wide range of hyperparameter configurations. This allowed us to identify the optimal settings that minimized the loss function, thereby enhancing the accuracy and predictive performance of our regression models.
2. MLP, LSTM, CNN: These neural network architectures were chosen for their ability to handle complex relationships within high-dimensional data, such as the biomechanical features extracted from trunk movement data. MLP excels in learning non-linear relationships, LSTM is adept at capturing temporal dependencies, and CNN is effective in extracting spatial features.
3. Random Forest Regressor: This ensemble method was selected due to its robustness in handling both categorical and continuous input data, its capability to detect interactions between variables, and its resilience against overfitting compared to individual decision trees.

### Preliminaries: *regression and classification techniques*

Table 2 presents a summary of regression and classification techniques used in the analysis of trunk movement in women with postnatal low back pain. These techniques employ machine learning models to predict low back pain and explore the relationship between trunk movement and pain symptoms. Each model is described along with its steps, equations, pros, and cons.

**Table 2:** Regression and classification techniques used.

Model	Description	Steps	Equations	Pros	Cons
Optimized Optuna^34^	A regression model that uses the Optuna framework to optimize hyperparameters and minimize the loss function	1. Define objective function 2. Use Optuna Regression to optimize hyperparameters 3. Sample different values from the search space 4. Run multiple trials 5. Retrieve best hyperparameters and evaluation metrics	minimize L(θ, h) where L is the loss function, θ represents learned regression model parameters, and h denotes hyperparameters to be optimized	-Efficiently explores hyperparameter space - Identifies optimal configuration for improved accuracy and predictive performance - Streamlines hyperparameter tuning process	- May require significant computational resources—May not be suitable for small datasets
MLP^35^	A neural network that uses multiple layers of interconnected nodes to learn complex relationships between input and output variables	1. Define MLP architecture 2. Split the dataset into training, validation, and test sets 3. Train MLP model on training data using backpropagation 4. Evaluate the model’s performance on the validation set 5. Adjust model architecture and hyperparameters until satisfactory performance is achieved 6. Evaluate the final model’s performance on the test set	y = f(W_2f.(W_1x + b_1) + b_2) where x is the input vector, W_1 and W_2 are weight matrices, b_1 and b_2 are bias vectors, f is the activation function, and y is the output	- Can learn complex relationships between input and output variables—Can handle high-dimensional input data—Can be used for both regression and classification tasks	- May require significant computational resources—May suffer from overfitting if not properly regularized
LSTM^36^	A recurrent neural network that can learn long-term dependencies by selectively retaining or forgetting information from previous time steps	1. Define LSTM architecture 2. Split the dataset into training, validation, and test sets 3. Train the LSTM model on training data using backpropagation through time 4. Evaluate the model’s performance on the validation set 5. Adjust model architecture and hyperparameters until satisfactory performance is achieved 6. Evaluate final model’s performance on test set	h_t, c_t = LSTM(x_t, h_{t-1}, c_{t-1}) where x_t is input at time step t, h_t is hidden state at time step t, c_t is cell state at time step t, and LSTM is the LSTM layer	- Can handle sequential data with long-term dependencies—Can learn complex temporal patterns—Can be used for both regression and classification tasks	- May require significant computational resources—May suffer from vanishing or exploding gradients if not properly regularized
CNN^37^	A neural network that uses convolutional layers to learn spatial features from input data such as images	1. Define CNN architecture 2. Split the dataset into training, validation, and test sets 3. Train CNN model on training data using stochastic gradient descent 4. Evaluate the model’s performance on the validation set 5. Adjust model architecture and hyperparameters until satisfactory performance is achieved 6. Evaluate the final model’s performance on the test set	y = f(W_5f.(P_4f.(C_3f.(P_2f.(C_1x + b_1) + b_2) + b_3) + b_4) + b_5) where x is the input tensor, C_1 to C_3 are convolutional layers, P_2 and P_4 are pooling layers, W_5 is the weight matrix, f is the activation function, and y is the output	- Can learn spatial features from input data such as images—Can handle high-dimensional input data—Can be used for both regression and classification tasks	- May require significant computational resources—May suffer from overfitting if not properly regularized
Random Forest Regressor^38^	An ensemble regression model that uses a combination of decision trees to make predictions	1. Define the number of decision trees and maximum depth of each tree 2. Split the dataset into training and test sets 3. Train the RandomForestRegressor model on training data 4. Evaluate the model’s performance on the test set	y = (1/N) * sum(f_i(x)) where N is the number of decision trees, f_i is the i-th decision tree, x is the input vector, and y is the output	- Can handle both categorical and continuous input data—Can handle missing data—Can detect interactions between variables	- May not perform well on high-dimensional data—May not perform well on datasets with skewed class distribution
SVR^39^	A regression model that uses support vector machines to find the hyperplane that maximizes the margin between predicted values and actual values	1. Define kernel function, regularization parameter, and other hyperparameters 2. Split the dataset into training and test sets 3. Train the SVR model on training data 4. Evaluate the model’s performance on testset	y = sum(alpha_i*y_i*K(x_i, x)) + b where alpha_i is the Lagrange multiplier, y_i is the target value, K is the kernel function, x_i is the input vector, x is the test input vector, and b is the bias term	- Can handle both linear and nonlinear input data—Can handle high-dimensional input data—Can be used for both regression and classification tasks	- May require significant computational resources—May not perform well on datasets with noisy or irrelevant features—May not perform well on datasets with imbalanced class distribution
Bagging Regressor^40^	An ensemble regression model that uses bootstrap aggregating to combine multiple decision tree models	1. Define the number of decision trees and maximum depth of each tree 2. Split the dataset into training and test sets 3. Train the BaggingRegressor model on training data using bootstrap aggregating 4. Evaluate the model’s performance on testset	y = (1/N) * sum(f_i(x)) where N is the number of decision trees, f_i is the i-th decision tree, x is the input vector, and y is the output	- Can handle both categorical and continuous input data—Can handle missing data—Can detect interactions between variables	- May not perform well on high-dimensional data—May not perform well on datasets with skewed class distribution—May not perform well on datasets with noisy or irrelevant

### The proposed framework

#### Dataset characteristics

The dataset consists of measurements related to postnatal low back pain, specifically related to the age, BMI, pain level, and range of motion (ROM) of participants. The data includes 70 observations, each with 9 columns.

- The dataset includes 9 columns of data, including Age, BMI, Pain, Flexion ROM, Extension ROM, RT Lateral flexion, LT Lateral flexion, Average, and Status_Average.
- The Age column represents the age of the participants in years.
- The BMI column shows the body mass index (BMI) of the participants in kg/m^2^.
- The Pain column represents the pain level reported by the participants on a scale of 0–10.
- The Flexion ROM column shows the range of motion (ROM) for the participants in flexion.
- The Extension ROM column shows the range of motion (ROM) for the participants in extension.
- The RT Lateral flexion column shows the range of motion (ROM) for the participants in right lateral flexion.
- The LT Lateral flexion column shows the range of motion (ROM) for the participants in left lateral flexion.
- The Average column represents the average of all the ROM values for each participant.
- The Status_Average column indicates whether the participant’s values were normal or abnormal.
- The dataset can be used to analyze the relationship between Age, BMI, and ROM values, as well as to predict the likelihood of experiencing postnatal low back pain based on these factors.

Table 3 presents descriptive statistics for each feature in the dataset, including the number of features, mean, median, standard deviation, minimum, 25th percentile, 50th percentile (median), 75th percentile, and maximum values.

**Table 3:** Descriptive statistics of the dataset features.

Category	Mean	Median	Std. Dev	Min	25%	50%	75%	Max
Status statistics	0.10	0.00	0.30	0.00	0.00	0.00	0.00	1.00
Age statistics	29.76	30.00	3.23	18.00	27.00	30.00	32.00	35.00
BMI statistics	27.39	27.70	2.40	23.20	25.50	27.70	29.10	36.80
Pain statistics	5.19	5.00	2.18	0.00	4.00	5.00	6.00	9.00
Flexion ROM stats	46.65	42.00	11.32	30.00	40.00	42.00	48.50	82.00
Extension ROM stats	14.27	14.00	3.41	10.00	12.00	14.00	16.00	25.00
RT Lat. flex. Stats	20.84	21.00	2.62	16.00	19.00	21.00	22.00	28.00
LT Lat. flex. Stats	21.25	21.00	2.15	18.00	20.00	21.00	23.00	27.00

**Figure 3:**
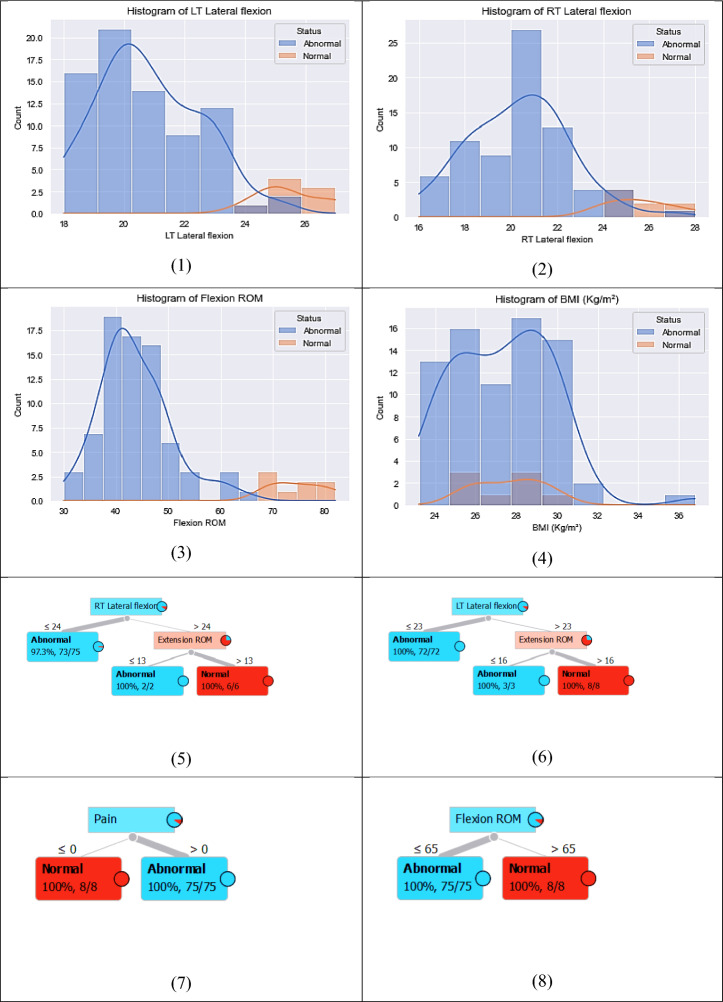
Correlation between dataset features.

Figure 3 shows the correlation between the dataset features.

Table 4 displays the correlation matrix of features within the dataset, providing valuable insights into the relationships between various attributes. The matrix presents the correlation coefficients, measured as Pearson’s R-values, indicating the strength and direction of the correlations. Each row and column in the table represents a specific feature, while the corresponding values in the cells represent the correlation coefficient between the two features. A correlation coefficient close to 1 signifies a strong positive correlation, indicating that the features tend to increase or decrease together. Conversely, a coefficient close to − 1 represents a strong negative correlation, suggesting an inverse relationship between the features. A coefficient near 0 indicates no significant correlation between the features. By examining the correlation matrix, researchers and readers can gain valuable insights into the interrelationships among the features, facilitating the identification of patterns, associations, or dependencies within the dataset.

**Table 4:** Correlation matrix of dataset features.

	Age	BMI	Pain	Flexion ROM	Extension ROM	RT Lateral flexion	LT Lateral flexion	Average
Age	1.0	0.285	0.097	− 0.023	0.070	0.121	0.026	0.037
BMI	0.285	1.0	− 0.067	0.091	0.041	0.026	0.118	0.089
Pain	0.097	− 0.067	1.0	− 0.732	− 0.532	− 0.389	-0.483	− 0.659
Flexion ROM	− 0.023	0.091	-0.732	1.0	0.662	0.453	0.612	0.957
Extension ROM	0.070	0.041	− 0.532	0.662	1.0	0.282	0.442	0.755
RT Lateral flexion	0.121	0.026	− 0.389	0.453	0.282	1.0	0.620	0.607
LT Lateral flexion	0.026	0.118	− 0.483	0.612	0.442	0.620	1.0	0.736
Average	0.037	0.089	− 0.659	0.957	0.755	0.607	0.736	1.0

**Figure 4:**
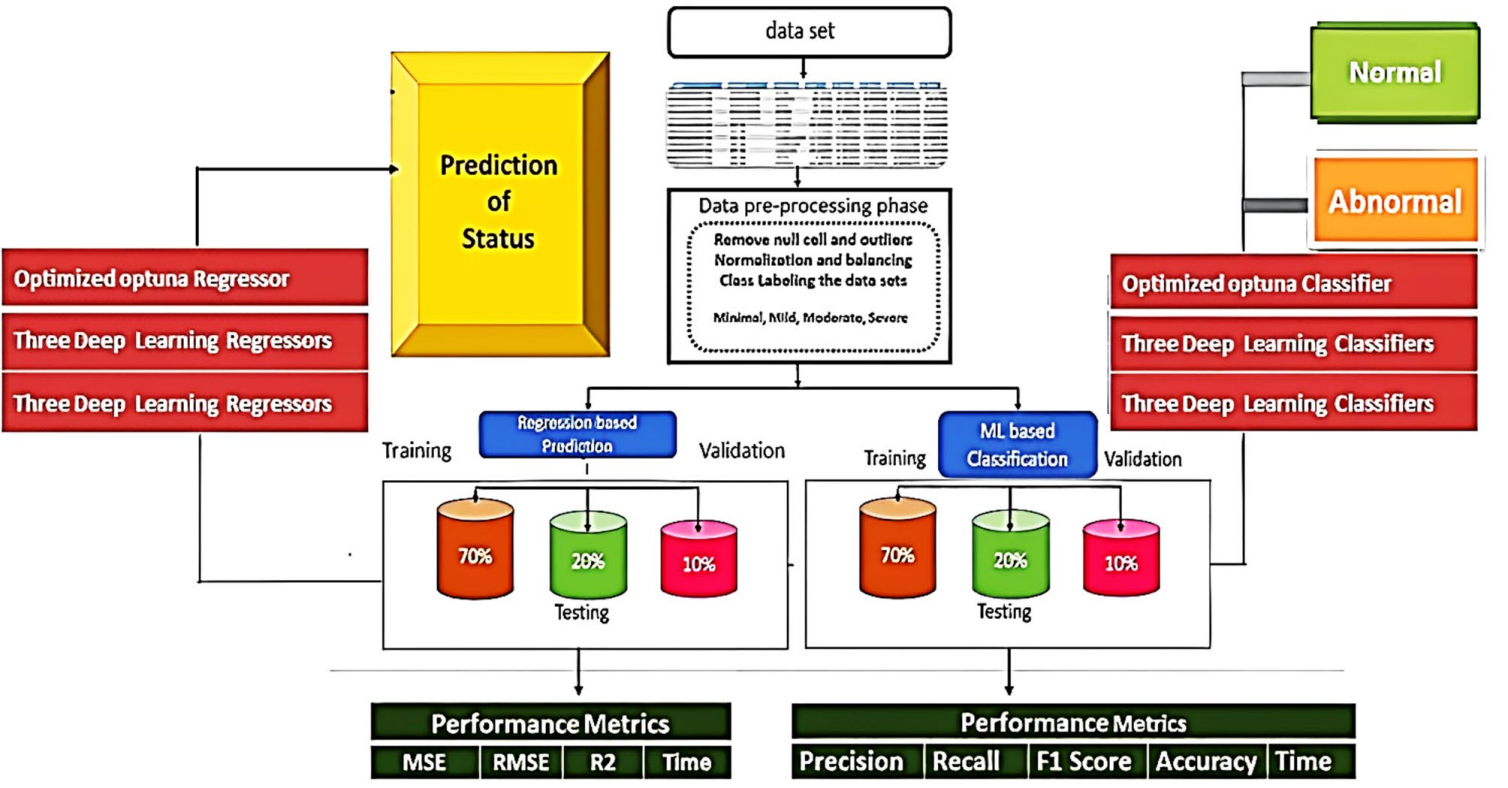
The general framework of the proposed prediction model.

To predict and classify the status of Pain and Flexion ROM, a machine learning framework was created. Figure 4 illustrates the general structure of the framework, which includes the prediction and classification process, as well as the performance metrics. The proposed classification and regression models are presented in the form of pseudocode in Figs. 4**, **Algorithm 1, and Algorithm 2 respectively.

Algorithm 1acts as a blueprint outlining the necessary steps for executing the suggested regression procedure. It begins with importing necessary modules such as pandas for loading and pre-processing dataset, Optuna for hyperparameter tuning, and KFold for cross-validation, among others. After loading the dataset with pandas, the preprocessing is started wherein features are separated and the target variable is encoded using LabelEncoder(). Later, the objective function for Optuna is defined and hyperparameters are optimized such as n_estimators, max_depth, min_samples_split, and min_samples_leaf. This is succeeded by the calculation of the mean squared error, and once optimal, a new RandomForestRegressor is built using the suggested hyperparameters. Algorithm 2, on the other hand, serves as the plan for the proposed classification procedure. It starts similar to the first algorithm by importing the necessary modules. After loading the dataset and separating the features and target variables, the data transforms the application of one-hot encoding and normalization. The dataset is then split into training and testing sets. An objective function for Optuna is defined and certain hyperparameters like max_depth and min_samples_split are optimized. The algorithm then moves on to optimizing the hyperparameters using Optuna and the best ones are selected for building a new DecisionTreeClassifier. This model is trained on the entire training set, after which predictions are made on the testing set. Finally, the performance of the model is evaluated and the best hyperparameters, accuracy, precision, recall, F1-score, and execution time are printed.

**Algorithm 1:**
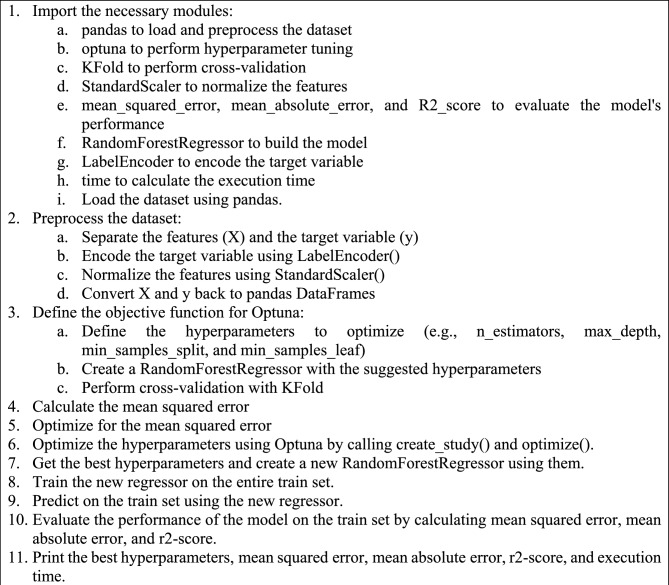
The pseudocode of the proposed regression model.

**Algorithm 2:**
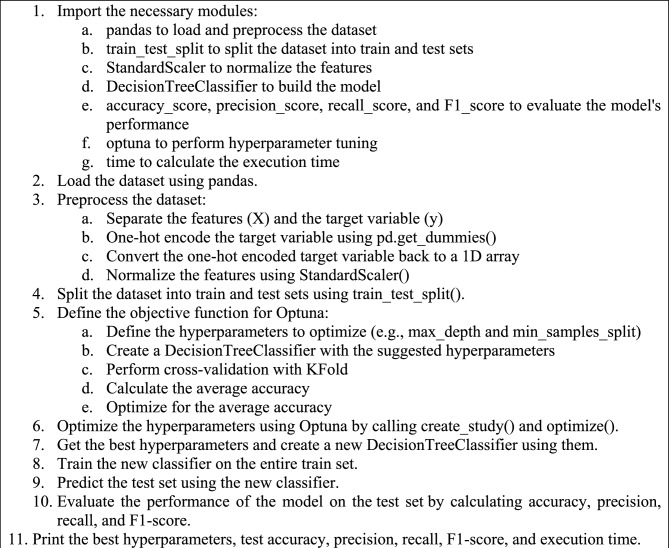
The pseudocode of the proposed classification model.

### Evaluation metrics for regression and classification models

#### Evaluation metrics for regression models

The determination coefficient R-square is one of the most common performances used to evaluate the regression model as shown in Eq. (1). On the other hand, the Minimum Acceptable Error (MAE) is shown in Eq. (2), while the Mean Square Error (MSE) is investigated in Eq. (3)^41–44^.

1 $${\text{R}}^{2}=\frac{\sum {\left(y-\dot{\widehat{y}}\right)}^{2}}{\sum {\left(y-\dot{\overline{y}}\right)}^{2}}$$

2 $$\text{MAE}=\frac{\sum_{i=1}^{n}\left|\widehat{{y}_{i}}-y\right|}{\text{n}}$$

3 $$\text{MSE}=\frac{\sum_{i=1}^{n}\left|\widehat{{y}_{i}}-{y}_{i}\right|}{\text{n}}$$

where y is the actual value, $\dot{\widehat{\text{y}}}$ is the corresponding predicted value, $\dot{\overline{\text{y}}}$ is the mean of the actual values in the set, and ***n*** is the total number of test objects^45^.

#### Evaluation metrics for classification models

Equations (4), (5), (6), and (7) are determined by the confusion matrix performance that represents the accuracy, precision, recall, and F1-score, respectively^46,47^.

4 $$\text{Accuracy}=\frac{\text{TP }+\text{ TN}}{\text{TP }+\text{ FP }+\text{ TN }+\text{ FN}}$$

5 $$\text{Precision}=\frac{\text{TP }}{\text{TP }+\text{ FP}}$$

6 $$\text{Recall}=\frac{\text{TP }}{\text{TP }+\text{ FN}}$$

7 $$\text{F}1 -\text{ score }=2* \frac{\left(\text{Precision }\times \text{ Recall}\right)}{\left(\text{Precision }+\text{ Recall}\right)}$$

These metrics are based on a “confusion matrix” that includes true positives (TP), true negatives (TN), false positives (FP), and false negatives (FN)^48^.

### Informed consent

Informed consent was obtained from all individual participants included in the study”.

## Results and analysis

To evaluate the effectiveness of our machine learning framework, we conducted experiments in this section. The experiments were performed on a computer with a 3 GHz i5 processor, 8 GB main memory, and a 64-bit Windows 10 operating system. We used the Python programming language to experiment.

### The results of the proposed regression machine learning technique

**Figure 5:**
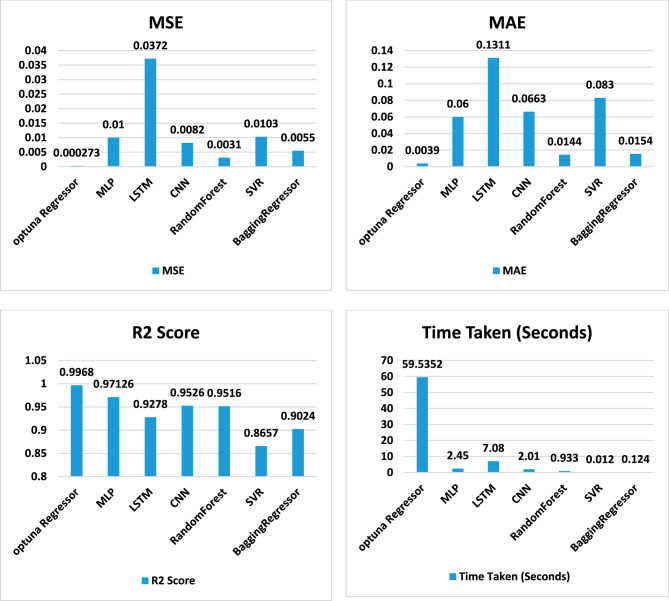
The performance metrics of the regression models.

Table 5 and Fig. 5 display the outcomes of a regression task conducted using various machine-learning models. The following is a detailed analysis and exposition of the table’s contents:

**Table 5:** The evaluation of different regression models to assess their performance.

Model	MSE	MAE	R2 score	Time taken (seconds)
Optimized optuna Regessor	0.000273	0.0039	0.9968	59.53
MLP	0.01	0.06	0.97126	2.45
LSTM	0.0372	0.1311	0.9278	7.08
CNN	0.0082	0.0663	0.9526	2.01
RandomForestRegressor	0.0031	0.0144	0.9516	0.9334
SVR	0.0103	0.0830	0.8657	0.0125
BaggingRegressor	0.0055	0.0154	0.9024	0.1249

- Model: This column provides the names of each machine learning model that was utilized in carrying out the regression task.
- MSE (Mean squared error): This column indicates the average of the squared deviations between the actual and predicted values. A smaller MSE value suggests superior performance.
- R2 score: This column reveals the coefficient of determination, which gauges the percentage of variability in the target variable that can be attributed to the predictors. A higher value of the R2 Score is indicative of excellent performance.
- Time taken (seconds): This column showcases the duration required for each model to fulfill the regression task.

As shown in Table 5:

- The Optimized optuna Regressor outperforms other models in terms of MSE, MAE, and R2 Score. It achieves the lowest MSE and MAE values, indicating better accuracy in predicting the target variable. Additionally, it achieves a high R2 Score of 0.9968, indicating that the model captures a significant amount of variance in the data. However, it also takes the longest time to train, with a duration of 59.53 s.
- Among the other models, the CNN and Random Forest Regressor also perform well with relatively low MSE, MAE, and high R2 Score values. They provide a good balance between accuracy and training time, with the CNN model being slightly faster to train than the Random Forest Regressor.
- On the other hand, the SVR and Bagging Regressor models show relatively higher MSE, MAE, and lower R2 Score values compared to the top-performing models. These models may not capture the underlying patterns in the data as effectively.
- The Optimized optuna Regressor, CNN, and Random Forest Regressor demonstrate the best performance in terms of accuracy while considering the trade-off with training time.

### The results of the proposed classification machine learning technique

**Figure 6:**
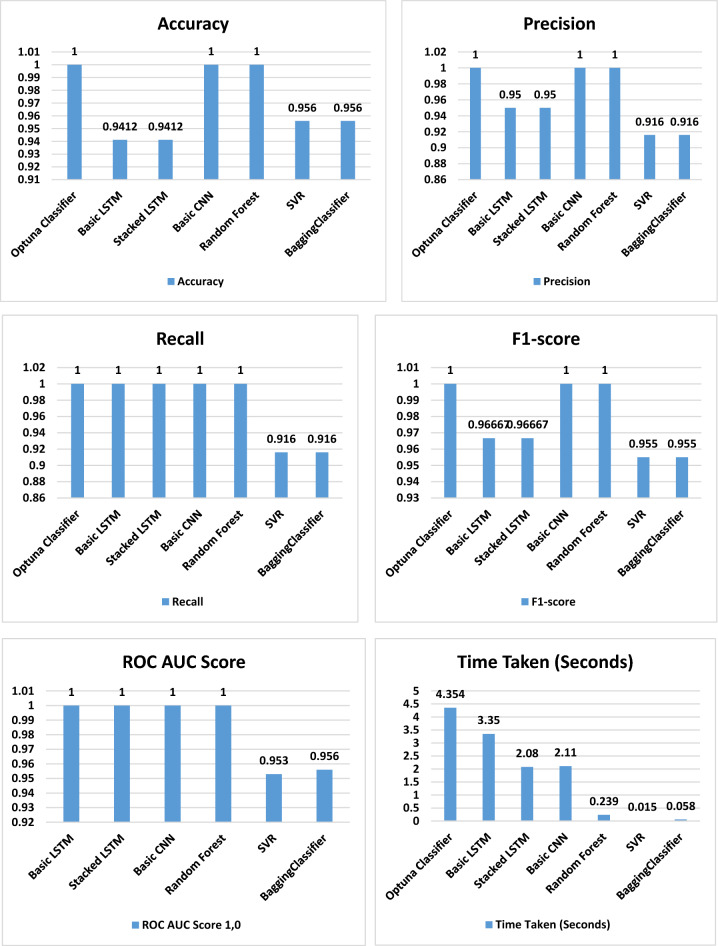
The performance metrics of the classification models.

Table 6 and Fig. 6 delineate the results garnered from a classification task conducted using various machine-learning models. Further insight and explanation of each column in the table are as follows:

**Table 6:** The performance metrics of different classification models.

Model	ROC AUC Score	Accuracy	Precision	Recall	F1-score	Time taken
Optimized optuna Classifier	1,0	1.0	1.0	1.0	1.0	4.3542
Basic LSTM	1.0	0.9412	0.95	1.0	0.96667	3.35
Basic Stacked LSTM	1.0	0.9412	0.95	1.0	0.96667	2.08
Basic CNN	1.0	1.0	1.0	1.0	1.0	2.11
RandomForestClassifier	1.0	1.0	1.0	1.0	1.0	0.239
SVR	0.9523	0.9565	0.9167	0.9167	0.9552	0.015
BaggingClassifier	0.956	0.9565	0.9167	0.9167	0.9552	0.058

- Model: This column furnishes the names of each machine-learning model that was put into use during the classification task.
- ROC AUC Score: This column signifies the Receiver Operating Characteristic (ROC) Area Under the Curve (AUC) score. This metric is a measure of the model’s aptitude in differentiating between the positive and negative classes. A superior ROC AUC score is an indicator of better performance.
- Accuracy: This column denotes the percentage of samples accurately classified. A higher accuracy value infers enhanced performance.
- Precision: This column reveals the ratio of true positive samples within all samples predicted as positive. A loftier precision score suggests a reduced number of false positives.
- Recall: This column displays the ratio of true positive instances out of the entire actual positive instances. A superior recall outcome refers to fewer false negatives.
- F1-score: This column indicates the harmonic mean of precision and recall. A higher F1 score embodies a better balance between the dynamics of precision and recall.
- Time Taken: This column exhibits the duration spent by each model to carry out the classification task completely.

From Table 6 and Fig. 6, we find that:

- Optuna and Basic CNN and Random Forest Classifier models are the top-performing against the selected metrics of ROC AUC Score, Accuracy, Precision, Recall, and F1 score, indicating their superior performance in this particular task.
- There’s an identical performance seen in Basic LSTM and Basic Stacked LSTM models in terms of ROC AUC Score, Accuracy, Precision, Recall, and F1 score.
- The SVR and BaggingClassifier models, with lower scores in ROC AUC, Accuracy, Precision, Recall, and F1 score, seem less effective for this task when compared to the others.
- The Optimized optuna Classifier was the slowest, taking 4.3542 seconds to complete the task.
- While, Basic LSTM, Basic Stacked LSTM, and Basic CNN models took considerable time, ranging from 2.08 to 3.35 seconds, they exceeded Random Forest Classifier, SVR, and Bagging Classifier models in terms of execution time.
- The Random Forest Classifier was the quickest with a computation time of only 0.239 seconds, whereas SVR and Bagging Classifier models completed the task in 0.015 seconds and 0.058 seconds, respectively.

The analysis reveals that while several models achieve perfect or near-perfect classification performance, considerations around computational time and the balance between precision and recall might dictate model choice depending on application priorities. For applications requiring both high accuracy and efficiency, the RandomForestClassifier stands out, while for scenarios where computational time is less of a concern, the Optimized Optuna Classifier or Basic CNN could be preferable due to their perfect classification scores.

### Feature correlations

Feature correlation is employed to discern the intensity and orientation of the linear association between two variables^41^. In the realm of regression models, the understanding of feature correlations is multi-purpose:

- Feature Selection: The process of dissecting the correlation between elements and the target variable lets us recognize features that manifest the most potent relationships with the target. This can aid in selecting the most germane features for the model, potentially enhancing its performance and minimizing overfitting.
- Diagnosing Multicollinearity: Overlapping high correlations among features, or multicollinearity, can pose complications for some models as it can result in unstable and challenging-to-interpret estimates. Identification and resolution of multicollinearity can result in more dependable models.
- Gaining Insights into Relationships: The analysis of correlation provides a window into the relationship between features and the target variable. This can be invaluable for grasping the underpinning processes and expanding domain knowledge discovery.
- Model Simplification: High correlations between two features might allow for the use of only one of them, doing away with any loss of major predictive power, simplifying the model, and reducing computation time.
- Enhancing Model Accuracy: By comprehending the relationships between features, engineered new features can better encapsulate the underlying patterns in the data, potentially enhancing the model’s accuracy.

**Figure 7:**
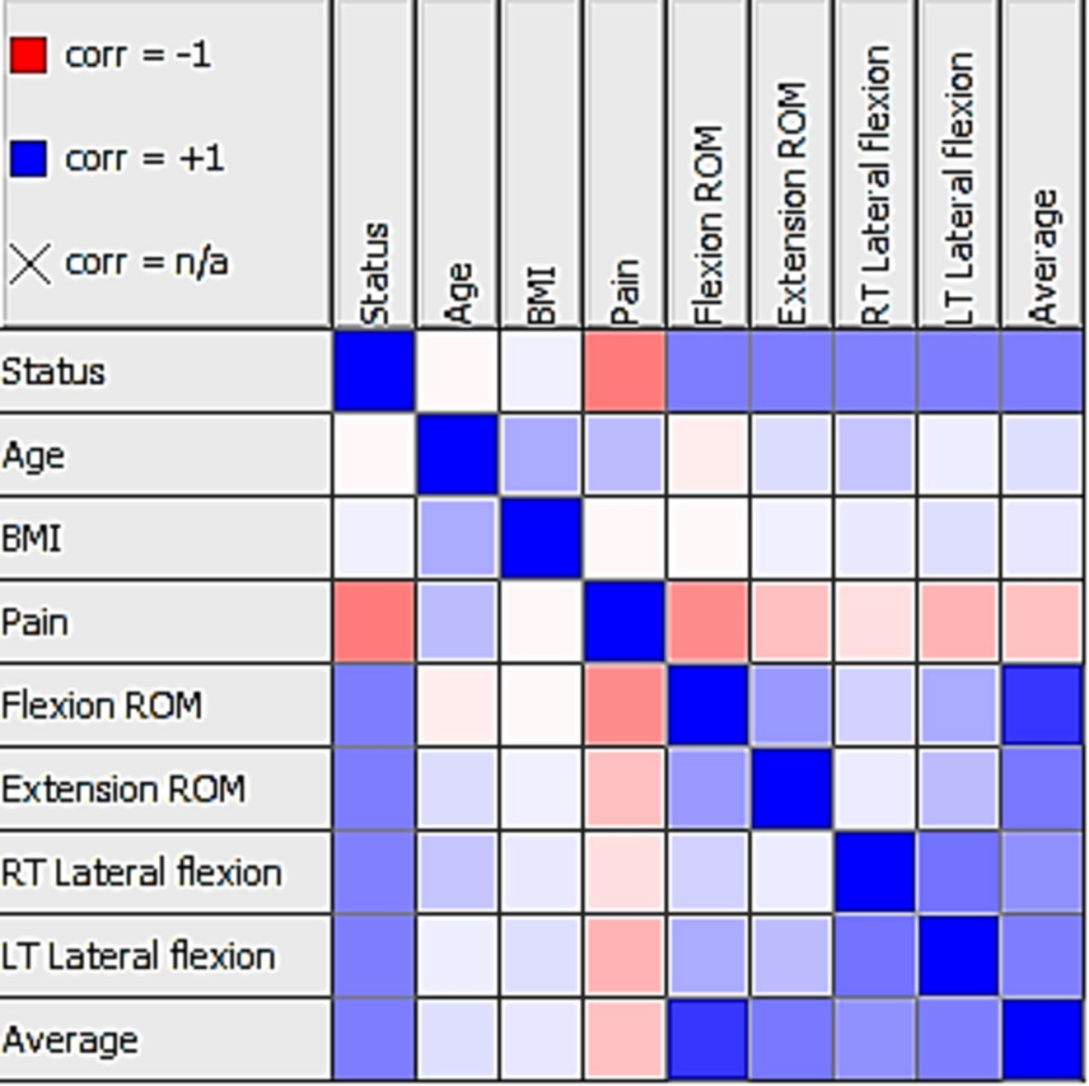
The heatmap correlation of the features.

Figure 7 lays out the heatmap correlation coefficient between distinct features within a dataset. The heatmap is structured in a manner that eases the understanding of how different features interrelate.

A heatmap is a data visualization tool that uses a color gradient to represent a matrix of values. In essence, it depicts how different variables are related by encoding the magnitude of the relationship in color intensity. A coefficient blue color of one points to a perfect positive correlation, implying that as one feature gets a boost, the other follows suit. On the flip side, a red color signifies a perfect negative correlation; an increase in one feature correlates with a decrease in another.

Based on the Fig. 7:

- The features showcasing the strongest positive correlation coefficients include Average Flexion ROM, Average Extension ROM, Average LT Lateral Flexion, and Average RT Lateral Flexion.
- The highest negative correlation coefficient is exhibited between features like Flexion ROM and Pain, and Extension ROM and Pain.
- Features indicating the lowest correlation coefficients include Age with Flexion ROM, Age with RT Lateral Flexion, and Age with BMI.
- The positive correlation between Average Flexion ROM with Average Extension ROM, and between Average LT Lateral Flexion with Average RT Lateral Flexion, alludes that a rise in one of these features typically corresponds to an increase in the other feature.
- The negative correlation observed between Flexion ROM and Pain, and between Extension ROM and Pain, implies that an upswing in one feature usually leads to a downturn in the other.
- The minuscule correlation coefficients linked to Age and Flexion ROM, Age and RT Lateral Flexion, and Age and BMI suggest the absence of a solid linear relationship between these elements.

The negative correlation between Flexion ROM and Pain in our study can be understood from the perspective of how these factors work within the human body. Normally, pain in the body often hinders free and flexible movements. When someone is experiencing pain, their instinctive response is to limit movement to prevent further discomfort or potential escalation of the injury. In the same way, with increased levels of pain, flexion, or the ability to bend joints resulting in a decrease of angle (for instance, bending the elbow), decreases. As pain increases, patients are less able to bend their body parts and therefore the range of motion decreases. That’s the reason why there’s a negative correlation between Pain and Flexion ROM as observed in this study. However, it is worth mentioning that our understanding is based on the general behavior observed in this study and there can be exceptions based on the individual’s pain tolerance, medical condition, and other factors.

### Feature selection

The selection of feature selection algorithms hinges on their aptitude to pinpoint the most pertinent features that enhance a model’s predictive potential. This in turn boosts the model’s performance, simplifies it, and improves ease of interpretation. Each feature selection strategy has distinct benefits and is chosen in accordance with specific prerequisites:

- F-value Selector: This approach picks features using F-statistics derived from ANOVA tests, gauging the significance of each feature. It’s well-suited for identifying linear relationships between features and the target variable.
- Mutual Information Selector: This method assesses mutual dependence between variables using information gain. It proves adept in identifying any form of statistical dependence, not just linear, which makes it an effective tool for feature selection.
- RFE with Logistic Regression: Recursive Feature Elimination (RFE) operates by recursively eradicating the least significant features based on model weights. When combined with logistic regression, it proves particularly effective for binary classification problems.
- Variance Thresholding: This method omits features whose variance doesn’t fulfill a specific threshold. It’s a straightforward baseline strategy for feature selection that aims to exclude features that are constant or nearly constant, given that they do not contribute to the model’s predictive accuracy.
- RFE with Random Forests: This is comparable to RFE with logistic regression, but employs Random Forests, an ensemble learning method. This pairing is resistant to overfitting and apt at identifying non-linear feature interactions.
- Feature Importance with Random Forests: The technique uses Random Forests to assign a rank to features based on their importance, which is determined by how much they reduce the impurity of the splits. This method proves useful in understanding feature contributions in complex datasets.

Table 7 represents the features chosen utilizing diverse feature selection strategies. The table analysis is explained as:

- Feature selection technique: This column exhibits the name of the feature selection method employed to select the features.
- Selected Features: This column displays the names of the features chosen by the feature selection method.

**Table 7:** Feature selection techniques and the most important features.

Feature selection method	Selected features
F-value selector	[‘Pain ‘, ‘Flexion ROM’, ‘Extension ROM’, ‘LT Lateral flexion’, ‘Average’]
Mutual information selector	[‘Pain ‘, ‘Flexion ROM’, ‘Extension ROM’, ‘LT Lateral flexion’, ‘Average’]
RFE with logistic regression	[‘Pain ‘, ‘Flexion ROM’, ‘Extension ROM’, ‘RT Lateral flexion’, ‘Average’’]
Select from the model with random forests	[‘Pain ‘, ’Flexion ROM’, ‘Extension ROM’, ‘Average’]
Variance thresholding	[‘Age’, ‘BMI’, ‘Pain ‘, ‘Flexion ROM’, ‘Extension ROM’, ‘RT Lateral flexion’, ‘LT Lateral flexion’, ‘Average’]
RFE with random forests	[‘Pain ‘, ‘Flexion ROM’, ‘Extension ROM’, ‘RT Lateral flexion’, ‘Average’]
Feature importance with random forests	[‘Flexion ROM’, ‘Average’, ‘Extension ROM’, ‘Pain ‘, ‘LT Lateral flexion’]

Based on the Table 7:

- The F-value selector, mutual information selector, and variance thresholding methods all selected the same set of features: Pain, Flexion ROM, Extension ROM, LT Lateral flexion, and Average.
- The RFE with logistic regression and RFE with random forests methods both selected Pain, Flexion ROM, Extension ROM, RT Lateral flexion, and Average, but excluded LT Lateral flexion.
- The Select from model with random forests method selected Pain, Flexion ROM, Extension ROM, and Average, but excluded LT Lateral flexion and RT Lateral flexion.
- The Feature importance with random forests method selected Flexion ROM, Average, Extension ROM, Pain, and LT Lateral flexion.
- The features selected by multiple methods (Pain, Flexion ROM, Extension ROM, LT Lateral flexion, and Average) may be the most important in the dataset.
- The features selected by only one or a few methods may still be important in the classification task but may have been overlooked by other methods.

## Discussion and future directions

Low back pain (LBP) and/or pelvic girdle pain (PGP) have a prevalence of 20–90% in the pregnant population, while a small number of women may suffer from a combination of both pains^9,10^. In this study, we found that the highest positive correlation coefficients are Average Flexion ROM Average Extension ROM, Average LT Lateral Flexion, and Average RT Lateral Flexion. The features with the highest negative correlation coefficient are Flexion ROM and Pain, and Extension ROM and Pain. The features with the lowest correlation coefficients are Age and Flexion ROM, Age and RT Lateral Flexion, and Age and BMI. It can explained by having persistent back pain was not found to be an important impetus for women to seek care postpartum, but a delay in becoming active again^49^. A decreased activity level can lead to disability, which is closely related to fear of movement in patients with chronic lower back pain, Biomechanical and hormonal changes from pregnancy are largely reversed by 3 months postpartum; consequently, it is assumed that other factors might interfere with recovery and explain the disability level postpartum^25^. The study and development of advanced machine learning techniques for accurate prediction and classification of lumbar range of motion in postnatal low back pain represent a significant advancement in the field of postnatal health. This innovative approach holds great potential for improving the assessment and management of postpartum women, allowing for more personalized and effective fitness interventions.

One of the key advantages of utilizing machine learning algorithms is their ability to process large amounts of data and extract patterns and correlations that might not be immediately apparent to human observers. In the context of postnatal health, this can be particularly valuable as it enables the analysis of various factors that contribute to abdominal fat thickness, including demographic information, lifestyle choices, genetic predispositions, and medical history^50^. By accurately predicting and classifying lumbar region ROM, machine learning models can provide important insights into the risk factors associated with LBP in postpartum women^51,52^. This can aid in identifying individuals who are at a higher risk of developing health complications. Early identification of these high-risk individuals can facilitate targeted interventions and preventive measures to mitigate potential health risks^53^, ^54^.

Furthermore, the utilization of advanced machine learning techniques in postpartum fitness can lead to more personalized and tailored fitness recommendations^55^. By considering an individual’s unique characteristics and history, machine learning models can provide specific exercise, dietary recommendations, and lifestyle modifications that are most likely to be effective in reducing postnatal LBP. This personalized approach has the potential to improve adherence to fitness programs and ultimately enhance the overall success rate in achieving postnatal women’s health goals.

The use of advanced machine learning techniques for accurate prediction and classification of lumbar region ROM in postnatal women represents a significant breakthrough in the field of postnatal health^56^. This technology has the potential to revolutionize the way we approach postnatal health, providing more accurate and personalized recommendations for women looking to regain their pre-pregnancy bodies. Another important benefit of this technology is its ability to provide personalized recommendations for postnatal women’s health. By analyzing a woman’s individual health and fitness data, including her age, weight, and other key metrics, machine learning algorithms can provide tailored recommendations for exercise routines, dietary changes, and other lifestyle modifications that can help women achieve their postnatal women’s health more quickly and effectively.

The results of the regression task reveal that the Optimized optuna Regressor excels in terms of accuracy, as evidenced by its lowest MSE and MAE values alongside a high R2 Score. This suggests that the model not only predicts the target variable with high precision but also captures a substantial proportion of the data’s variance. However, this superior performance comes at the cost of computational time, as it takes significantly longer to train than other models. For applications where prediction accuracy is paramount and computational resources are not a limiting factor, the Optimized optuna Regressor emerges as the preferred choice. The CNN and RandomForestRegressor models offer a balanced alternative, providing competitive accuracy with shorter training times. Their lower MSE and MAE values coupled with high R2 Scores make them suitable for scenarios requiring a trade-off between accuracy and efficiency. Among these, the CNN model stands out for its slightly faster training speed, making it a viable option for real-time applications or large-scale datasets. On the other hand, the SVR and BaggingRegressor models lag behind in performance metrics, with higher MSE and MAE values and lower R2 Scores. This indicates that these models struggle to effectively capture the underlying patterns in the data, making them less desirable for tasks where accuracy is crucial. Nonetheless, they could be considered for preliminary analyses or when dealing with smaller datasets where training time is less of a concern.

In the classification task, the Basic CNN and RandomForestClassifier models emerge as the frontrunners, achieving perfect scores across all performance metrics. This includes the highest ROC AUC Score, Accuracy, Precision, Recall, and F1-score, demonstrating their exceptional capability in distinguishing between positive and negative classes. Their performance is unparalleled, making them ideal for critical applications where misclassification errors must be minimized. The Basic LSTM and Basic Stacked LSTM models exhibit identical performance metrics, suggesting that stacking additional LSTM layers does not necessarily improve the model’s performance in this context. Both models are outperformed by the Basic CNN and RandomForestClassifier but remain viable options due to their acceptable performance and moderate training times. The SVR and BaggingClassifier models, with their lower performance scores, are less effective for classification tasks, particularly when high accuracy is essential. However, their quick computation times might make them suitable for rapid prototyping or when dealing with very large datasets where time efficiency is a priority.

The feature correlation analysis reveals that certain features, such as Average Flexion ROM and Average Extension ROM, are strongly positively correlated, indicating that they tend to move in the same direction. This suggests that these features may be redundant, and using one instead of the other could potentially simplify the model without sacrificing predictive power. Similarly, the negative correlation between Pain and Flexion ROM/Extension ROM implies that higher pain levels are associated with reduced range of motion, aligning with clinical observations. The weak correlations involving Age suggest that age may not be a strong predictor of the target variable in this dataset. This finding is crucial for feature selection, as it allows for the exclusion of Age from the model if computational efficiency is a concern.

The feature selection techniques applied to the dataset highlight several key features that are consistently identified as important across different methods. Pain, Flexion ROM, Extension ROM, and Average are frequently selected, indicating their critical role in predicting the target variable. The inclusion of these features in the final model is likely to improve its predictive accuracy. However, the discrepancy in the selected features across different methods underscores the need for careful consideration when choosing a feature selection strategy. Features like RT Lateral flexion and LT Lateral flexion, which are selected by some methods but not others, may still hold valuable predictive information that should not be disregarded solely based on one method’s output.

### Future directions

Machine Learning-Based Analysis of Trunk Movement in Women with Postnatal Low Back Pain is an area of research that has the potential to improve our understanding of the causes and treatments of low back pain in postpartum women. Here are some future directions that can be explored in this area of research:

1. Larger datasets: The performance of machine learning models is often improved with larger datasets. Future studies can collect larger datasets to increase the accuracy and robustness of the models and to enable more complex analyses.
2. Longitudinal studies: Longitudinal studies that follow women over time can provide insights into the progression of postnatal low back pain and the effectiveness of different treatments. Machine learning models can be used to analyze changes in trunk movement patterns over time and to predict the likelihood of developing low back pain in the future.
3. Integration of multiple data sources: In addition to trunk movement data, other data sources such as electromyography (EMG) and electroencephalography (EEG) can be used to provide a more comprehensive understanding of the mechanisms underlying postnatal low back pain. EEG allows the recording of the electrical activity of the brain. In the context of our research, its implication lies in the fact that pain, including low back pain, is a subjective experience that is processed in the brain. Therefore, understanding the brain’s reaction to pain signals, reflected in its electrical activity, could supply us with valuable insights about the subjective experience of pain and its neural correlations. Moreover, adaptations in motor control strategies in response to back pain, such as modifications in movement patterns, are guided by the central nervous system whose activity can be studied using EEG. Hence, combining the data from trunk movement analysis with EEG readings could confront the complicated nature of postnatal low back pain from both peripheral (trunk movement) and central (brain activity) perspectives, potentially creating a ‘holistic’ picture of this intricate issue. Machine learning models can be used to integrate and analyze data from multiple sources for a more holistic approach to diagnosis and treatment.
4. Personalized treatment: Machine learning models can be used to develop personalized treatment plans based on the specific trunk movement patterns and other factors unique to each individual. This can improve the effectiveness of treatments and reduce the risk of adverse effects.
5. Transfer learning: Transfer learning is a technique that allows machine learning models to transfer knowledge learned from one task to another. Future studies can explore the use of transfer learning to improve the accuracy and efficiency of machine learning models for analyzing trunk movement in women with postnatal low back pain.
6. Clinical implementation: The ultimate goal of this research is to improve the diagnosis and treatment of postnatal low back pain in clinical settings. Future studies can explore the feasibility and effectiveness of implementing machine learning-based analyses of trunk movement in clinical practice to improve patient outcomes.

The use of machine learning to analyze trunk movement in women with postnatal low back pain is a promising area of research with many potential future directions. By collecting larger datasets, conducting longitudinal studies, integrating multiple data sources, developing personalized treatment plans, using transfer learning, and implementing machine learning in clinical practice, researchers can improve our understanding of postnatal low back pain and develop more effective treatments.

## Limitations

While our approach, involving the Machine Learning-Based Analysis of Trunk Movement in Women with Postnatal Low Back Pain, holds potential in understanding and managing low back pain in postpartum women, several limitations manifest. These limitations include limited generalizability, limited interpretability, limited data availability, limited feature selection, limited causal inference, and limited clinical implementation.

### These limitations can be summarized as follows

1. Limited generalizability: Recognizing the restricted applicability of our models beyond our research demographic, we’ve carefully used our findings to guide clinical application instead of formulating rigid rules.
2. Limited interpretability: To counter this ‘black box’ nature of machine learning models, we systematically documented the variable importance during the model training phase. This allows for some transparency on which input variables significantly influenced our results.
3. Limited data availability and overfitting: We were concerned about a limited sample size potentially leading to overfitting. As a resolution, we used techniques like cross-validation and regularization during the model training phase, which helps minimize overfitting, and we’ve also emphasized the need for further larger-scale studies before broad clinical application.
4. Limited feature selection: While developing the model, we selected features based on prior clinical knowledge and statistical significance to ensure that the features used were most likely to contribute to postnatal LBP.
5. Limited causal inference: We acknowledge that machine learning is more suited for prediction rather than identifying causal relationships. For causal inference, we plan to incorporate more traditional statistical models in the future.
6. Limited clinical implementation: Despite the potential benefits, we understand the hurdles stopping the widespread clinical implementation of these models. However, there are ongoing efforts at our end to improve the cost-effectiveness, and we’re also focusing on initiating proactive discourses with clinicians and patients about the positive aspects of this technology.

## Conclusions

This paper has conducted a comprehensive analysis of trunk movement in women experiencing postnatal low back pain (LBP) using advanced machine learning techniques. The primary objectives were to identify pivotal features associated with LBP and to construct precise predictive models. Machine learning approaches demonstrated significant promise in analyzing biomechanical factors pertinent to postnatal LBP. The study leveraged regression and classification algorithms on a dataset comprising 100 postpartum women, equally split between those with and without LBP. The Optimized Optuna Regressor achieved exceptional performance in regression tasks, yielding a mean squared error (MSE) of 0.000273, a mean absolute error (MAE) of 0.0039, and an R2 score of 0.9968. In classification tasks, both the Basic CNN and Random Forest Classifier achieved outstanding accuracy metrics, including an area under the receiver operating characteristic curve (AUC) of 1.0, precision of 1.0, recall of 1.0, and F1-score of 1.0, outperforming alternative models. Key predictive features identified through the study included pain (correlation of -0.732 with flexion range of motion), range of motion measures (correlation of 0.662 with flexion and extension), and average movements (correlation of 0.957 with flexion). Feature selection consistently highlighted pain intensity, flexion and extension range of motion, lateral flexion, and average movement as influential factors across various analytical methods. While acknowledging the limitations inherent in an initial dataset and considerations of generalizability, the application of machine learning provided valuable quantitative insights. The models demonstrated robust performance in both regressing (MSE < 0.01, R2 > 0.95) and classifying (accuracy > 0.94) trunk biomechanics, effectively distinguishing characteristics related to LBP. Future studies could benefit from integrating additional demographic, clinical, and patient-reported factors to further refine personalized risk prediction and treatment strategies. This preliminary application of advanced analytics underscores the potential utility of machine learning in enhancing both the identification of LBP risk factors and the optimization of treatment outcomes. The findings from this study offer critical insights into employing machine learning techniques for analyzing trunk movement in postnatal women with low back pain, aiming to inform the development of more effective therapeutic interventions.

## Data Availability

The dataset and code used in this study are public and all test data are available at this portal (https://github.com/tarekhemdan/Trunk_Movement).
